# Characteristics of autologous protein solution and leucocyte-poor platelet-rich plasma for the treatment of osteoarthritis of the knee

**DOI:** 10.1038/s41598-020-67099-y

**Published:** 2020-06-29

**Authors:** Shiho Wasai, Masato Sato, Miki Maehara, Eriko Toyoda, Ryoka Uchiyama, Takumi Takahashi, Eri Okada, Yoshiko Iwasaki, Satoko Suzuki, Masahiko Watanabe

**Affiliations:** 1 Department of Orthopaedic Surgery, Surgical Science, Tokai University School of Medicine, Isehara, Kanagawa Japan; 2 Center for Musculoskeletal innovative Research and Advancement (C-MiRA), Tokai University, Graduate School of Medicine, Isehara, Japan

**Keywords:** Medical research, Osteoarthritis, Predictive markers, Risk factors

## Abstract

Recently, platelet-rich plasma (PRP) has received attention as a treatment for patients with osteoarthritis of the knee (OAK), a chronic degenerative disease, to bridge the gap between conservative and surgical treatments. Here, we investigated the differences in the humoral factors present in two types of PRP purified using the Autologous Protein Solution (APS) kit (group Z; leucocyte-rich PRP) or the Cellaid Serum Collection Set P type (group J; leucocyte-poor [LP]-PRP). Differences in humoral factors between healthy subjects (n = 10) and OAK patients (n = 12; group Z = 6, group J = 6), and the relationship between humoral factors and clinical outcome scores were investigated. Both anti-inflammatory and inflammatory cytokines were highly enriched in APS. The concentrations of tumour necrosis factor (TNF)-α, platelet-derived growth factor, fibroblast growth factor, soluble TNF-receptor 2, soluble Fas and transforming growth factor-β1 were higher in group Z, while the total amounts were higher in group J. The concentration of interleukin-1 receptor antagonist was positively correlated with the magnitude of change in the clinical outcome score and may contribute to improving knee-joint function. This is the first description of the humoral factors in APS and LP-PRP prepared from healthy subjects or OAK patients of Asian descent.

## Introduction

Osteoarthritis of the knee (OAK) is a chronic degenerative disease that progresses slowly with age. There are estimated to be 25.3 million patients with OAK in Japan. The number of symptomatic patients is estimated at 8 million and is expected to continue to increase with Japan’s hyper-aging population^1^.

Current methods of treatment for patients with OAK include patient education, exercise, oral treatments (i.e. non-steroidal anti-inflammatory drugs [NSAIDs], acetaminophen, tramadol), conservative therapy by intra-articular injection of hyaluronic acid or steroids or surgical treatments, including arthroscopic debridement of the knee joint, osteotomies around the knee such as high tibial osteotomy, unicompartmental knee arthroplasty (UKA) and total knee arthroplasty (TKA)^2,3^.

In recent years, cell therapies and platelet-rich plasma (PRP) have been investigated as treatments to bridge the gap between conservative and surgical treatments. Intra-articular injection of PRP for the treatment of OAK has been reported to relieve pain, suppress inflammation and regenerate cartilage^4–7^. A variety of commercial kits for preparing PRP are available, and in Japan, the use of PRP therapy is gaining popularity. The blood components and humoral factors of PRP vary significantly depending on the preparation methods used, and the leucocyte content is used to categorize PRPs as pure PRP, leucocyte-poor PRP (LP-PRP), or leucocyte-rich PRP (LR-PRP). More recently, autologous protein solution (APS), a version of LR-PRP with a higher concentration of humoral factors, has received some attention.

Recent studies have investigated the humoral factors associated with OAK, including those present in PRPs prepared from healthy donors and/or from OAK patients of European or American descent^8^. However, to our knowledge, there have been no previous reports concerning the humoral factors contained in PRPs prepared from healthy or OAK patients of Asian descent.

At Tokai University, we have begun to offer PRP treatment for knee disorders as a non-insured medical treatment approved under a provisional plan for class II regenerative medicine in accordance with the Act to Ensure the Safety of Regenerative Medicine in Japan. We have offered two types of PRP: that prepared using the APS kit (Zimmer Biomet, Warsaw, IN, USA), and that prepared using the Cellaid Serum Collection Set P type (JMS, Hiroshima, Japan), categorized as LR-PRP and LP-PRP, respectively. In July 2018, we conducted a test run using healthy subjects, and in September 2018, we began offering PRP treatment to OAK patients.

In the present study, we investigated the differences in the humoral factors and blood components between PRPs prepared from healthy subjects and OAK patients using the two kits. The relationship between the levels of humoral factors and clinical outcome scores was also investigated.

This study was reviewed and approved by the Institutional Review Board of the Tokai University School of Medicine (18R-134) and conducted in compliance with relevant guidelines. Written informed consent was obtained from all participants.

## Results

The characteristics of 10 healthy subjects and 12 OAK patients are shown in Table 1. There were no significant differences in the characteristics of the two groups of OAK patients.

**Table 1:** Characteristics of healthy subjects and OAK patients.

		Healthy subjects	OAK patients	
			group Z	group J
n		10	6	6
Female		5	5	4
Male		5	1	2
Age (years; mean ± standard deviation)		38.6 ± 11.0	72.5 ± 19.7	70.7 ± 8.1
K–L classification	2		2	0
	3		3	5
	4		1	1

K-L classification: Kellgren-Lawrence classification

### Blood cell analysis

The whole blood total cell count did not differ significantly between healthy subjects and OAK patients (either group Z or J). We also compared the levels of red blood cells (RBC), platelets (PLT) and white blood cells (WBC) contained in PRPs prepared by the different kits or from healthy subjects compared with OAK patients. Comparison of the kits indicated that RBC and WBC were significantly higher in group Z than in group J for both healthy subjects and OAK patients, but PLT did not differ significantly (Table 2).

**Table 2:** Blood cell analysis of each group. Whole blood (WB), APS, LP-PRP.

	WB			APS		LP-PRP	
	Healthy	Group Z	Group J	Healthy	Group Z	Healthy	Group J
RBC (×10^4^/μl)	438.84 ± 46.44	402.60 ± 68.94	445.11 ± 76.44	250.67 ± 3.14	172.38 ± 61.96	3.14 ± 1.62	5.18 ± 7.74
PLT (×10^4^/μl)	18.80 ± 5.99	18.47 ± 3.81	16.03 ± 3.26	127.03 ± 95.49	83.82 ± 54.34	95.49 ± 40.64	49.89 ± 17.19
WBC (×10^2^/μl)	56.84 ± 20.25	48.90 ± 12.38	59.97 ± 6.38	530.01 ± 0.74	428.88 ± 138.06	0.74 ± 0.76	28.38 ± 54.82
PLT concentration ratio (%)				667 ± 342	495 ± 356	529 ± 256	308 ± 129
WBC concentration ratio (%)				963 ± 388	911 ± 319	1.54 ± 2.11	50.0 ± 86.0

Data reported as mean ± standard deviation.

The increase in concentration of PLT in the PRPs compared with that in whole blood was slightly higher for group Z than for group J in both healthy subjects (group Z: 667 ± 342%; group J: 529 ± 256%) and OAK patients (group Z: 495 ± 356%; group J: 308 ± 129%), but the difference was not significant.

The ratio of WBC concentration in PRPs to that in whole blood was significantly higher in group Z than in group J, although the WBC concentration was lower in group J for both healthy subjects (group Z: 963 ± 388%; group J: 1.54 ± 2.11%) and OAK patients (group Z: 911 ± 319%; group J: 50.0 ± 86.0%).

### Analysis of humoral factors

**Figure 1:**
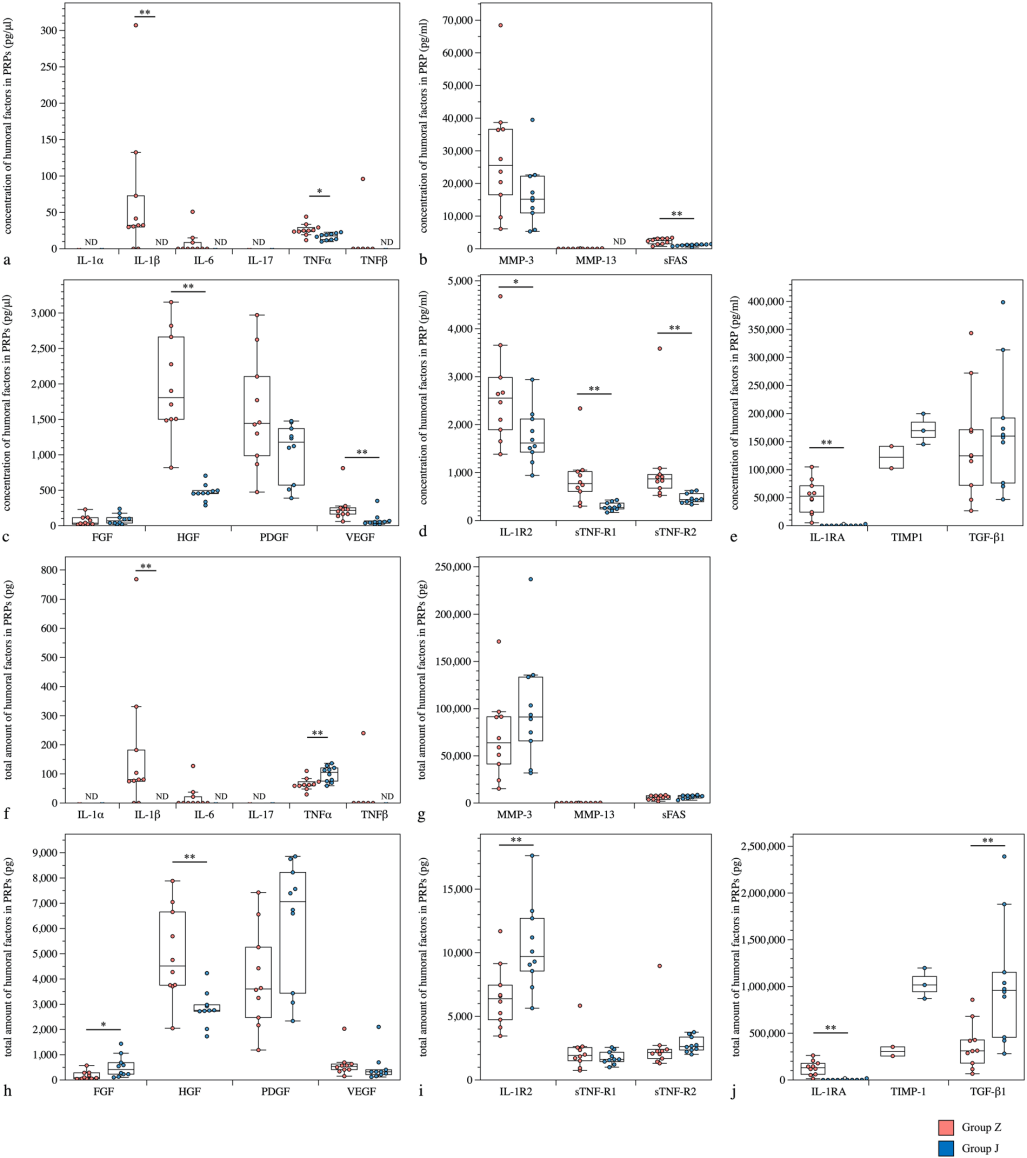
Comparison of humoral factors in PRPs from groups Z and J of healthy subjects. (**a–e**) The concentration of humoral factors in PRPs from groups Z and J of healthy subjects. (**f–j**) The total amount of humoral factors in PRPs from groups Z and J of healthy subjects. *P < 0.05, **P < 0.01. ND, non-detectable concentration of humoral factors using ELISA. Values above the upper detection limit were not included in the calculations, and the adjusted n numbers are as follows: TIMP-1 of group Z, n = 2; TIMP-1 of group J, n = 3.

**Figure 2:**
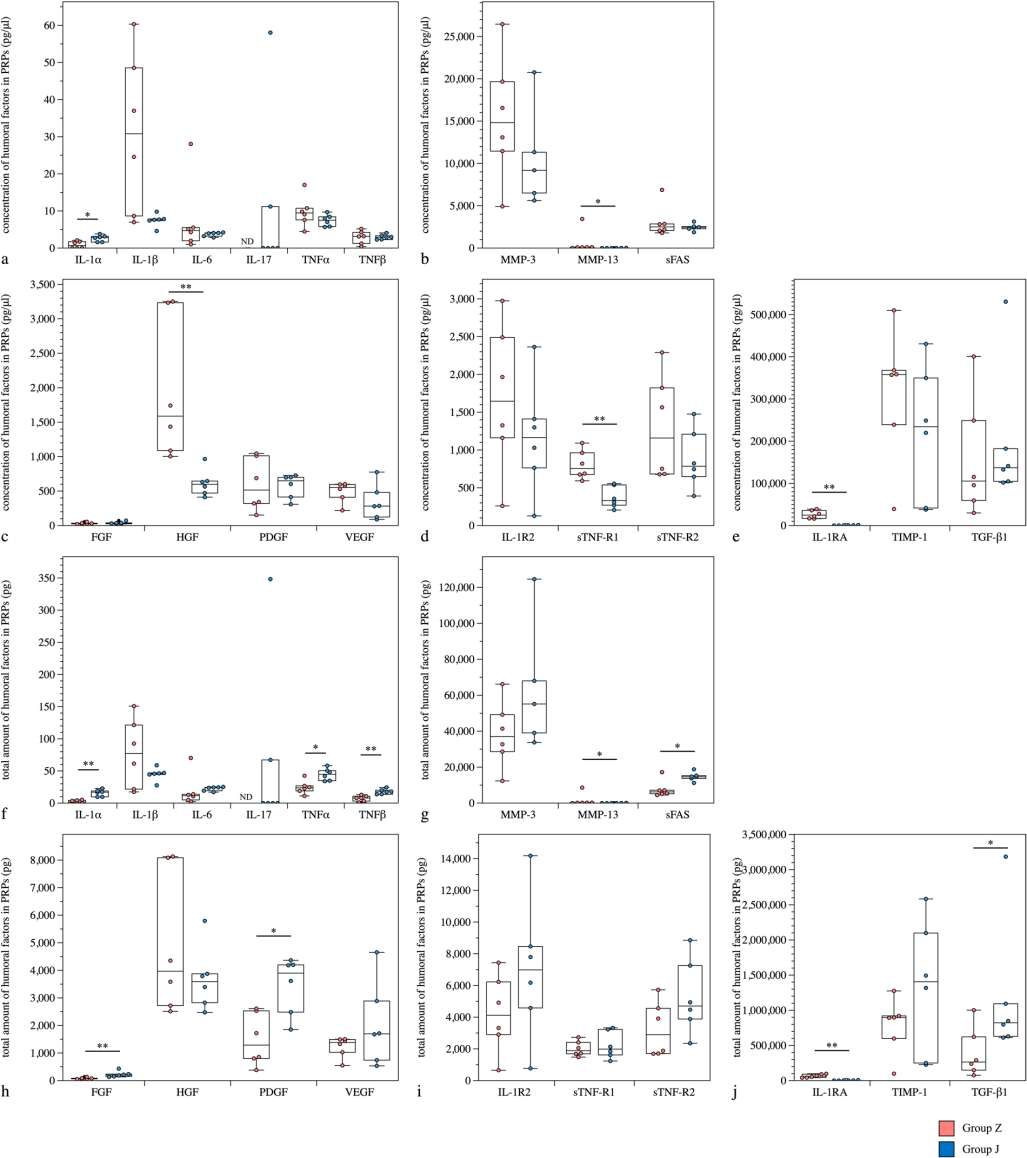
Comparison of humoral factors in PRPs produced from groups Z and J of OAK patients. (**a–e**) Concentration of humoral factors in PRPs from groups Z and J of OAK patients. (**f–j**) Total amount of humoral factors in PRPs from groups Z and J of OAK patients. *P < 0.05, **P < 0.01. ND, non-detectable concentration of humoral factors using ELISA. Values above the upper detection limit were not included in the calculations, and the adjusted n numbers are as follows: MMP-3 of group J, n = 5.

The comparison between kits showed that in healthy subjects, the concentrations of interleukin (IL)-1β, IL-1 receptor 2 (IL-1R2), IL-1 receptor antagonist (IL-1RA), tumour necrosis factor (TNF)-α, hepatocyte growth factor (HGF), vascular endothelial growth factor (VEGF), soluble TNF-receptor 1 (sTNF-R1), sTNF-R2 and soluble Fas (sFas) were all significantly higher in group Z than in group J. When the total amounts of each humoral factor contained in the PRPs were calculated, the total amounts of IL-1β, IL-1RA and HGF were significantly higher in group Z, and the total amounts of IL-1R2, TNF-α, PDGF, FGF, sTNF-R2, sFas and TGF-β1 were significantly higher in group J (Fig. 1). In OAK patients, the concentrations of IL-1RA, HGF, sTNF-R1 and matrix metalloproteinase (MMP)-13 were significantly higher in group Z, and only IL-1α was significantly higher in group J. However, the total amount of IL-1RA and MMP-13 contained in the APS of group Z was significantly higher than that in group J, whereas the total amounts of IL-1α, TNF-α, TNF-β, platelet-derived growth factor (PDGF), fibroblast growth factor (FGF), sTNF-R2, sFas and transforming growth factor (TGF)-β1 were higher in group J (Fig. 2).

**Figure 3:**
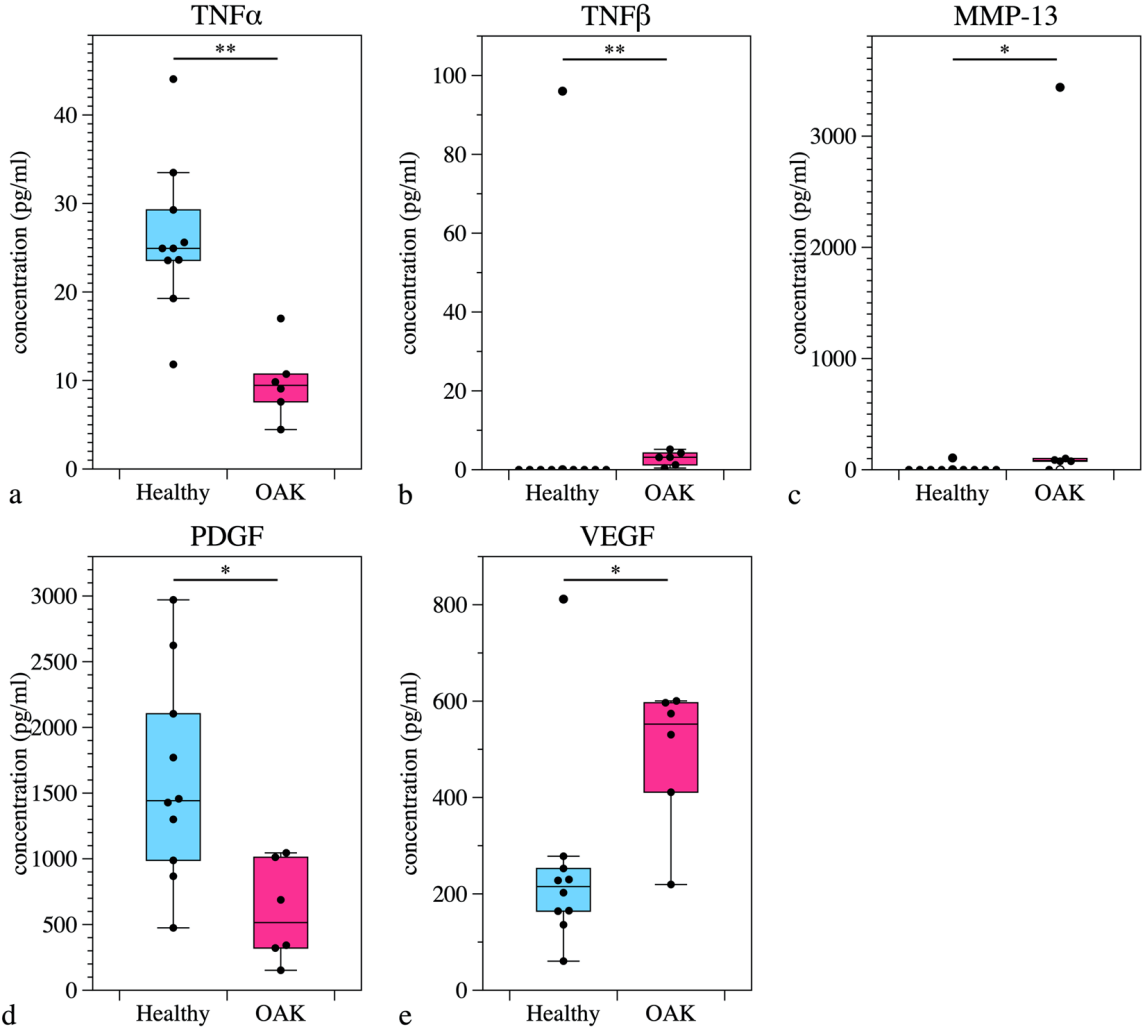
(**a–e**) Comparison of humoral factors in PRP prepared from healthy subjects and OAK patients in group Z. Significant differences were detected in the concentrations of TNF-α, TNF-β, PDGF, VEGF and MMP-13. The plots show median values (middle lines), interquartile ranges (boxes), 1.5 IQR (whiskers) and outliers (dots). *P < 0.05, **P < 0.01 comparing healthy subjects and OAK patients.

The separate comparisons between healthy subjects and OAK patients of groups Z and J indicated that there were significant differences between controls and patients of group Z in the concentrations of TNF-α, TNF-β, VEGF, PDGF and MMP-13. The concentrations of TNF-α and PDGF were significantly higher in healthy subjects than in OAK patients, and the concentrations of VEGF and MMP-13 were significantly higher in OAK patients (Fig. 3).

**Figure 4:**
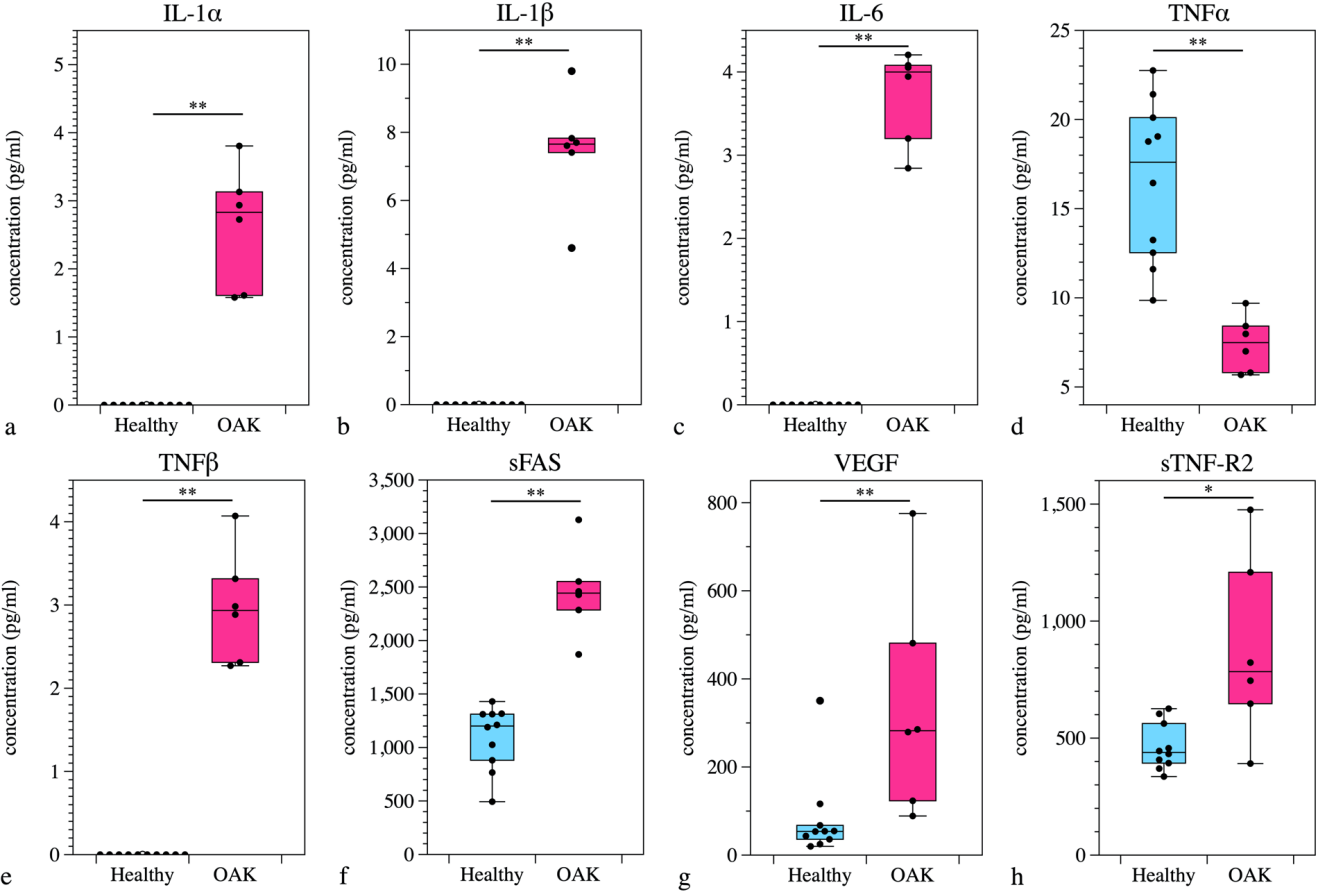
(**a–h**) Comparison of humoral factors in PRP prepared from healthy subjects and OAK patients in group J. Significant differences were detected in the concentrations of IL-1α, IL-1β, IL-6, TNF-α, TNF-β, sFas, VEGF and sTNF-R2. The plots show median values (middle lines), interquartile ranges (boxes), 1.5 IQR (whiskers) and outliers (dots). *P < 0.05, **P < 0.01 comparing healthy subjects and OAK patients.

In group J, significant differences between controls and OAK patients were detected in the concentrations of IL-1α, IL-1β, IL-6, TNF-α, TNF-β, VEGF, TNF-R2 and sFas: the concentration of TNF-α was higher in controls, and the concentrations of the other factors were higher in OAK patients (Fig. 4).

### Examination of clinical outcome scores

**Figure 5:**
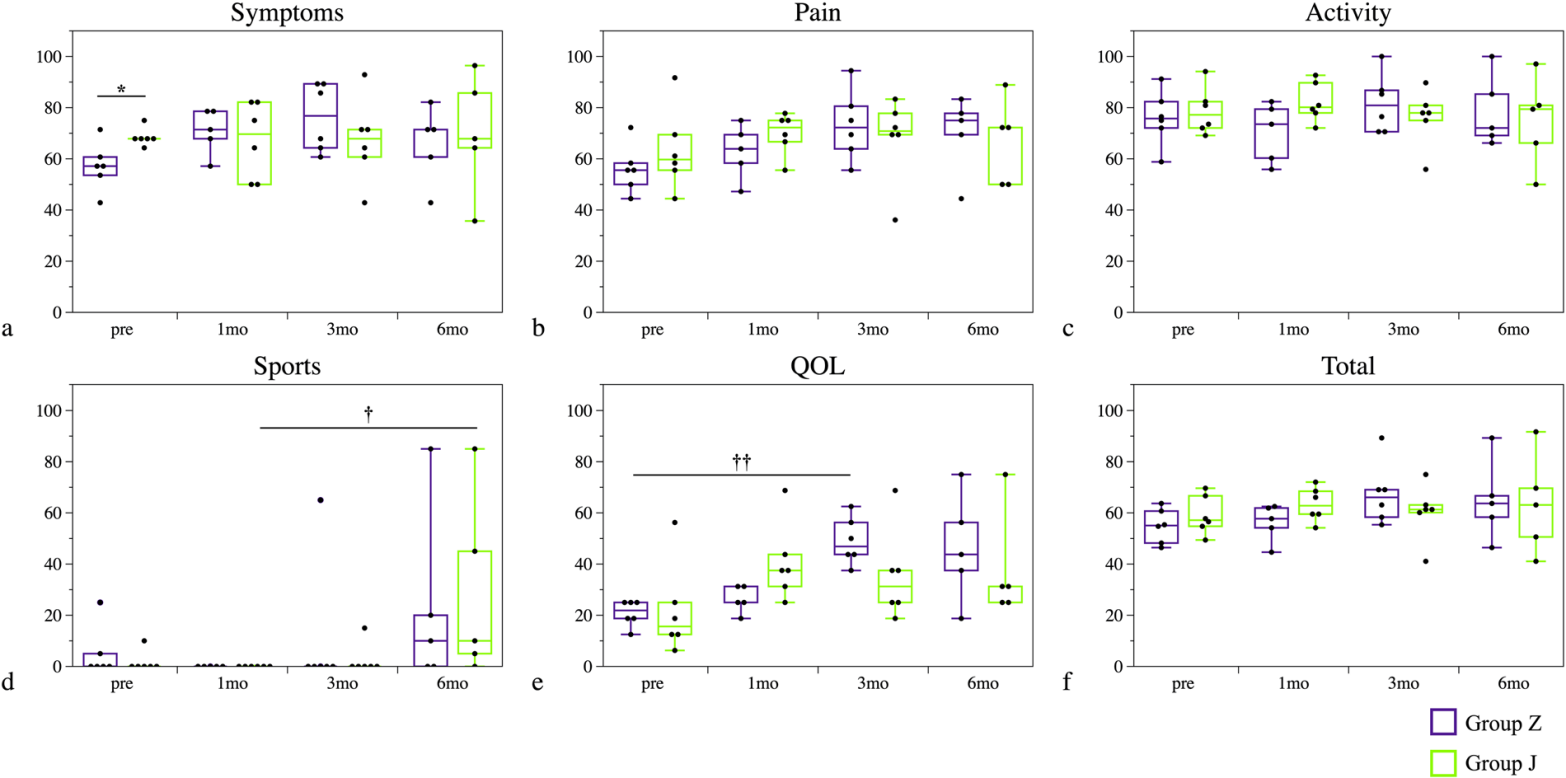
KOOS sub-scores and KOOS total score. Symptoms sub-score before PRP treatment differed significantly between groups Z and J. In group Z, the QOL sub-score at 3 months improved significantly compared with the sub-score before PRP treatment (*P* = 0.007). In group J, sports sub-scores improved significantly between 1 and 6 months after treatment (*P* = 0.020). The plots illustrate median values (middle lines), interquartile ranges (boxes), 1.5 IQR (whiskers) and outliers (dots). *indicates significant difference between group Z and group J. ^†^indicates difference between follow-up time points in each group. *P < 0.05, **P < 0.01, ^†^*P* < 0.05, ^††^*P* < 0.01.

**Figure 6:**
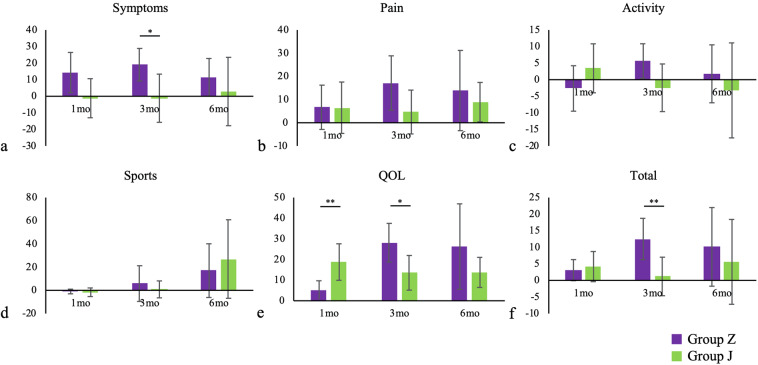
Magnitude of change from preoperative scores for KOOS sub-scores and KOOS total score. Magnitudes of changes in symptoms sub-scores, QOL sub-scores and KOOS total scores at 3 months were significantly higher in group Z, while the magnitude of change for QOL sub-scores at 1 month was significantly higher in group J. This figure shows mean ± standard deviation. *P < 0.05, **P < 0.01.

The Knee injury and Osteoarthritis Outcome Score (KOOS) (symptoms, pain, activity, sports and quality of life [QOL] sub-scores and total scores) of both groups before PRP treatment, and at 1, 3 and 6 months after treatment are presented in Figs. 5 and 6.

The symptoms sub-scores before PRP treatment differed significantly between groups, but there were no significant differences in the other sub-scores or the total scores. However, for group Z, QOL sub-scores at 3 months improved significantly compared with the QOL sub-scores before PRP treatment (*P* = 0.007), while for group J, significant improvement was observed in the sports sub-scores between 1 and 6 months after treatment (*P* = 0.020).

The results of the analysis of the correlations between KOOS total score and either the concentration or the total amount of humoral factors in PRPs are summarized in Table 3. Significant correlations were detected between the following: the KOOS total score at 1 month after treatment and the concentrations of IL-1β, TNF-α, sTNF-R1, IL-6 and sFas; the KOOS total score at 3 months and the concentration of PDGF; and the KOOS total score before treatment and the total amount of PDGF.

**Table 3:** Correlations between KOOS total score or magnitude of change in KOOS total score and the concentration or total amount of the 17 different humoral factors measured in PRPs.

	KOOS total score								Magnitude of change in KOOS total score					
	Concentration				Total amount				Concentration			Total amount		
	pre	1 mo	3 mo	6 mo	pre	1 mo	3 mo	6 mo	1 mo	3 mo	6 mo	1 mo	3 mo	6 mo
IL-1α	− 0.244	−0.309	−0.595	−0.622	−0.048	0.073	−0.536	−0.483	−0.307	−0.629	−0.604	0.000	−0.705*	−0.536
IL-1β	− 0.168	−0.649*	0.281	0.231	−0.030	−0.553	0.192	0.233	−0.530	0.516	0.340	−0.674*	0.283	0.287
IL-1R2	− 0.194	−0.222	−0.192	−0.013	0.221	0.402	−0.212	0.186	−0.258	−0.096	0.038	0.070	−0.469	0.089
IL-1RA	− 0.080	−0.323	0.464	0.247	−0.058	−0.306	0.470	0.253	−0.133	0.689*	0.290	−0.144	0.679*	0.294
TNF-α	− 0.513	−0.634*	−0.268	−0.389	0.064	0.248	−0.371	−0.267	−0.362	0.069	−0.239	0.037	−0.550	−0.356
TNF-β	− 0.387	−0.080	−0.401	−0.504	0.084	0.466	−0.389	−0.208	0.329	−0.215	−0.372	0.381	−0.592*	−0.251
HGF	− 0.297	−0.515	0.030	−0.129	−0.125	−0.358	−0.182	−0.214	−0.436	0.288	−0.047	−0.601	−0.140	−0.241
PDGF	0.500	0.374	0.640*	0.094	0.581*	0.546	0.110	−0.007	−0.061	0.440	−0.158	−0.090	−0.338	−0.281
TIMP-1	− 0.144	−0.244	0.208	−0.223	0.159	0.153	−0.040	−0.250	−0.078	0.399	−0.152	−0.067	−0.186	−0.290
VEGF	− 0.075	−0.208	0.288	0.025	0.101	0.187	−0.007	−0.038	−0.145	0.449	0.024	0.091	−0.094	−0.093
FGF	0.481	0.296	0.245	0.340	0.430	0.422	−0.175	0.013	−0.178	−0.073	0.280	−0.071	−0.595*	−0.073
sTNF-R1	−0.338	−0.691*	0.200	−0.069	−0.018	−0.217	−0.211	−0.350	−0.469	0.550	0.032	−0.422	−0.268	−0.460
sTNF-R2	0.220	0.042	0.436	−0.164	0.330	0.241	−0.031	−0.298	−0.192	0.402	−0.378	−0.222	−0.318	−0.545
IL-6	− 0.353	−0.606*	−0.133	−0.099	−0.130	−0.335	−0.259	−0.119	−0.467	0.116	0.079	−0.465	−0.239	−0.021
sFAS	−0.453	−0.683*	−0.204	0.028	0.049	0.092	−0.407	0.007	−0.404	0.105	0.294	−0.077	−0.587*	0.051
TGF-β1	− 0.147	−0.309	−0.124	−0.174	0.098	0.037	−0.166	−0.077	−0.359	−0.043	−0.123	−0.221	−0.305	−0.121
MMP-3	− 0.398	−0.433	−0.007	0.096	0.006	0.295	−0.222	0.092	0.111	0.369	0.365	0.470	−0.286	0.189

The correlation coefficients (r) between the two parameters are shown in this table. *P < 0.05, **P < 0.01.

Correlations were analysed for scores before PRP treatment and those at 1, 3, and 6 months after PRP treatment.

In addition, Table 3 shows the coefficients of correlation between the magnitude of change in KOOS total score and either the concentration or the total amount of humoral factors in PRPs. Significant correlations were detected between the following: the magnitude of change in KOOS total score at 3 months and the concentrations of IL-1RA and sTNF-R1; the magnitude of change in KOOS total score at 1 month and the total amounts of IL-1β and HGF; and the magnitude of change in KOOS total score at 3 months and the total amounts of IL-1α, IL-1RA, TNF-β, FGF and sFas. However, it should be noted that these correlations are not strong.

## Discussion

Recent studies have reported associations between OAK and the levels of humoral factors such as the well-known inflammatory cytokines IL-1β and TNF-α. The activation of the nuclear factor (NF)-κB pathway by these inflammatory cytokines results in the production of MMPs and VEGF, initiating an inflammatory response. This inflammatory response can lead to synovial inflammation and cartilage degradation^9,10^. By contrast, the anti-inflammatory cytokines such as the receptor agonists IL-1RA, sTNF-R1, sTNF-R2 and growth factor TGF-β are well known for their cartilage-repairing activity^11,12^.

APS, which is prepared by concentration and enrichment of PRP by the addition of polyacrylamide beads, contains a higher concentration of humoral factors than LP-PRP^13^. In this study, APS prepared from both healthy subjects and OAK patients (group Z) contained a higher concentration of both anti-inflammatory cytokines and inflammatory cytokines compared with the LP-PRP of group J. Nevertheless, analysis of the total amount of these cytokines within APS or LP-PRP revealed that for both healthy subjects and OAK patients, the total amounts of TNF-α, PDGF, FGF, sTNF-R2, sFas and TGF-β1 were actually higher in group J, while the total amount of IL-1RA was higher in group Z. Thus, when evaluating the clinical efficacy of different types of PRP kits, it is important to consider both the concentration and the total amount of the different humoral factors.

A comparison between the PRPs produced from healthy subjects and OAK patients using each kit revealed that the levels of the inflammatory cytokine TNF-α were higher for healthy subjects, in contrast to previous reports analysing those produced from individuals of Europe and the United States^14^. The blood concentration of TNF-α has been reported to increase in dementia and type 2 diabetes, as well as with age^15,16^. The reason for the higher concentration in the small number of relatively young healthy subjects included in the present study is unclear, but the finding indicates the need for further studies.

There was a high concentration of VEGF in the PRPs prepared from OAK patients using both kits. In OAK patients, VEGF is a direct initiator of cartilage destruction and synovial inflammation, and our results suggest that systemic concentrations of VEGF are higher in OAK patients than in controls. We have previously reported that bevacizumab, an anti-VEGF antibody, has an inhibitory effect on OAK after both intravenous (systemic) and intra-articular (local) administration^17,18^. This suggests that the pathology of OAK is not only local to the knee joint but is also a systemic disease.

Clinical outcome scores evaluated using KOOS revealed a significant difference between kits only for the preoperative symptoms sub-score; no differences were detected in other sub-scores or KOOS total scores. However, the extent to which clinical outcome scores improved for other sub-scores and KOOS total scores over time was greater for group Z than for group J; of note, the magnitude of change in symptoms and QOL sub-scores and the KOOS total score at 3 months were significantly greater in group Z.

Kon *et al*. reported that compared with saline injection, APS injection significantly improved the Western Ontario and McMaster Universities Osteoarthritis Index (WOMAC) percentage pain score from baseline to 1 year after treatment, and currently, the recommended schedule of APS treatment is a single rather than multiple injections per year^19^. However, the present study showed that the magnitude of change in clinical outcome scores tended to be greatest at 3 months postoperatively, suggesting that APS injections every 3 to 6 months may be effective. Future studies to confirm this will require a greater number of participants who undergo 12 months of postoperative follow-up.

To examine the therapeutic effect of the concentration and total amount of humoral factors, we analysed the correlation between the KOOS and the concentration or the total amount of humoral factors contained in each PRP. The concentrations of six humoral factors and the total amount of one humoral factor were correlated with the KOOS total score, while the total amounts of six humoral factors and the concentration of one humoral factor were correlated with the magnitude of change in the KOOS total score.

Both the concentration and total amount of PDGF were positively correlated with KOOS total score, but not with the magnitude of change in the KOOS total score. This result suggests that the amount of PDGF contained in PRPs tends to be higher in patients with higher KOOS, but this may depend mainly on other factors such as age. Thus, a high level of PDGF may not necessarily be useful to predict the therapeutic effect of PRPs.

No correlation with KOOS total score was found for IL-1RA, which has attracted attention as a major anti-inflammatory cytokine present in APS that has a pain-suppressing effect, or for TGF-β1. However, both the concentration and the total amount of IL-1RA were significantly correlated with the magnitude of change in KOOS total score, and IL-1RA was the only factor that was positively correlated with the magnitude of change in KOOS total score. These results suggest that IL-1RA contained in APS and LP-PRP may have a positive therapeutic effect. However, the characteristics of the patients who produce high levels of IL-1RA in PRPs are unclear. The characteristics of responders and non-responders to IL-1RA are also unclear, and this warrants further investigation.

Humoral factors IL-1α, IL-1β, TNF-β, FGF and sFas were negatively correlated with the magnitude of change in the KOOS total score. Of note, IL-1β and sFas showed a significant negative correlation with both KOOS total score and the magnitude of change in KOOS total score.

IL-1β, which has various catabolic effects on chondrocytes, is associated with the induction of inflammatory cytokines such as IL-6, TNF and nitric oxide, up-regulation of aggrecanases and MMPs, down-regulation of extracellular matrix synthesis by reducing proteoglycan and collagen and cartilage surface dedifferentiation through various signalling pathways, including the NF-κB pathway^10,11,20^.

sFas is an apoptosis-related protein, and its blood concentration is known to be elevated in OAK and rheumatoid arthritis (RA) patients^21^. Similar results were obtained in our study. Cheng *et al*.^22^ reported that sFas is likely to inhibit Fas-mediated apoptosis and promote an autoimmune response in a mouse model of RA. It has been suggested that sFas accumulates in the joints of RA patients as a result of this inhibition of apoptosis, leading to prolonged inflammation and synovitis. sFas concentrations in the joints of OAK patients have been reported to be lower than those in the joints of RA patients^23^, but similar mechanisms may be responsible for inflammation in OAK patients.

As described above, IL-1β and sFas in PRPs have negative effects on clinical outcome scores, and our results suggest that patients with more IL-1β and sFas in their PRPs may have poor therapeutic responses and tend to have lower KOOS total scores.

The concentration of VEGF, which was higher in OAK patients than in healthy subjects, did not correlate with KOOS total score or the magnitude of change in KOOS total score.

MMP-3 and MMP-13 are known to be elevated in the synovium, synovial fluid and blood serum of OAK patients^14,24,25^, but in this study, the concentrations and total amounts of MMP-3 and MMP-13 contained in PRPs were not correlated with KOOS total score. It has previously been reported that WOMAC scores and concentration of MMP-13 in blood serum were weakly correlated, while WOMAC scores after TKA surgery were positively correlated with the concentration of MMP-13 within the synovial fluid^14^. An analysis of the concentrations of MMPs in synovial fluid prior to PRP treatment may be warranted. Furthermore, the levels of tissue inhibitor of metallopeptidase (TIMP)-1, an inhibitor of MMPs, did not differ significantly between PRPs prepared using the two kits and was not correlated with KOOS total score. Consideration of the relative levels of TIMPs and MMPs may be useful^26^.

Future studies should include a larger number of subjects and an analysis of the correlation of PRP contents with the findings of magnetic resonance imaging (MRI) techniques such as the Magnetic Resonance Observation of Cartilage Repair Tissue scoring system and the modified Noyes classification system, to determine the best type of PRP to use and the timing of treatment relative to the degree of OAK.

## Conclusion

In group Z, both anti-inflammatory and inflammatory cytokines were highly enriched in APS, and while the concentrations of TNF-α, PDGF, FGF, sTNF-R2, sFas and TGF-β1 were lower in group J than in group Z, the total amount was higher.

For the clinical outcome scores, there was no significant difference in the KOOS total scores of group Z and group J after PRP treatment. However, the magnitude of changes in symptoms and QOL sub-scores and KOOS total score at 3 months was significantly higher in group Z, while only the magnitude of change in the QOL sub-score at 1 month was significantly higher in group J.

There was a significant positive correlation between IL-1RA levels and the magnitude of change in the KOOS total score, suggesting that IL-1RA may contribute to the improvement of knee-joint function.

Further analysis of the relationship of PRP content with MRI findings including more subjects is necessary.

## Materials and Methods

Ten healthy subjects over the age of 20 without OAK were included in the test run conducted in July 2018 (Table 1). Two types of PRP were purified separately from each healthy subject using the APS kit (group Z) or the Cellaid Serum Collection Set P type (group J).

Twelve OAK patients who received PRP treatment between September 2018 and August 2019 at Tokai University Hospital were prospectively randomized into group Z or group J (Table 1). The inclusion criteria for OAK patients were as follows: age ≥ 20 years and experiencing knee pain; and Kellgren-Lawrence (K-L) classification of ≥ 2.

The exclusion criteria were as follows: positive for Hepatitis B surface antigen, hepatitis C virus antibodies, human immunodeficiency virus antibodies and/or *Treponema pallidum* antibodies; diagnosed with cancer; affected by a serious medical condition; using anti-cancer drugs, biological drugs or immunosuppressants; active infection; and a history of drug hypersensitivity.

### Purification of APS and LP-PRP

To prepare APS, 60 ml of blood (5 ml of anticoagulant citrate dextrose solution, formula A [ACD-A] + 55 ml of blood) was collected in a 60-ml syringe. The blood was injected into the APS Separator (Zimmer Biomet), which was then centrifuged at 745 ×*g* (3200 rpm) for 15 min using a GPSIII centrifuge. Six millilitres of the cell solution were extracted and transferred to an APS Concentrator (Zimmer Biomet). The device was then centrifuged at 291 ×*g* (2000 rpm) for 2 min using the same centrifuge, and approximately 2.5 ml of APS was collected from the device.

To prepare LP-PRP, 60 ml of blood (5 ml of ACD-A + 55 ml of blood) was used to prepare 6 ml of LP-PRP using three Cellaid Serum Collection Sets P type. The Cellaid Serum Collection Set P type has a structure in which the primary and secondary containers have a connecting tube. After putting the primary container and the secondary container in separate empty 50 ml centrifuge tubes, 20 ml of blood from the 60 ml syringe was injected into the primary container and centrifuged at 200 ×*g* for 15 min. The supernatant containing platelets and plasma was transferred to the secondary container, and then centrifuged at 1200 ×*g* for 15 min. Finally, excess plasma in the secondary container was returned to the primary container, and 2 ml of LP-PRP was recovered from the secondary container. The above procedure was repeated twice more to collect a total of approximately 6 ml of LP-PRP.

From each healthy subject, approximately 120 ml of blood was collected using two 60 ml syringes, from which 2.5 ml of APS and 6 ml of LP-PRP were prepared.

From each OAK patient, approximately 60 ml of blood was collected using a 60 ml syringe; from this, 2.5 ml of APS from patients allocated to group Z and 6 ml of LP-PRP from those allocated to group J were prepared as described above.

### Blood cell analysis

Excess samples were collected from whole blood, plasma, APS and/or LP-PRP of each healthy subject and OAK patient, and blood cell analysis was performed to determine the number and concentration of RBC, PLT and WBC (Table 2).

One healthy subject and one OAK patient from group Z were excluded from the blood cell analysis because of haemolysis and a lack of excess samples of APS, respectively.

### Analysis of humoral factors

Using the excess samples of PRPs, catabolic factors (IL-1α, IL-1β, IL-6, IL-17, TNF-α, TNF-β, MMP-3, MMP-13 and sFas), antagonists, inhibitors and decoy receptors of catabolic factors (IL-1RA, IL-1R2, sTNF-R1, sTNF-R2, and TIMP-1) and various growth factors (PDGF, FGF, TGF-β1, VEGF, and HGF) were measured by enzyme-linked immunosorbent assays (ELISA).

IL-1RA and TGF-β1 were measured using the respective single-sample ELISA kits: Human IL-1RA SimpleStep ELISA Kit (Abcam, Cambridge, MA, USA) and Human TGF-β1 Quantikine ELISA Kit (R&D Systems, Minneapolis, MN, USA). The other humoral factors were measured using Q-Plex ELISA multiplex arrays (Quansys Biosciences, Logan, UT, USA). We analysed a total of 19 humoral factors and calculated the concentration and total amount of each humoral factor in APS and LP-PRP.

The levels of humoral factors were compared between the PRPs produced by the two kits and between healthy subjects and OAK patients for each kit. Values that were below the detection limit were treated as zero, and values that were above the maximum detection limit were excluded (Figs. 1–4).

### Examination of clinical outcome scores

The clinical outcomes of the 12 OAK patients were evaluated by KOOS before PRP treatment and at 1, 3 and 6 months after treatment. The scores were compared over time and between the two groups (Fig. 5).

In addition, the magnitude of change in KOOS from that before PRP treatment as baseline was calculated at 1, 3 and 6 months after PRP treatment, and compared between the two groups (Fig. 6).

Correlation coefficients were calculated to examine the correlation between KOOS and each humoral factor (Table 3). IL-17 and MMP-13, which were below the detection limit in six or more subjects, were excluded.

### Statistical analysis

The significance of differences between groups was determined using the Mann–Whitney *U* test for non-normally distributed quantitative data. The Kruskal–Wallis test was used to detect significant differences between three groups or more.

Bivariate correlation analysis was performed using the Pearson’s test between KOOS and each humoral factor.

All statistical analyses were performed using SPSS software (v. 25.0; IBM, Armonk, NY, USA). *P* values of <0.05 were considered significant.
